# Genetic Code Evolution Reveals the Neutral Emergence of Mutational Robustness, and Information as an Evolutionary Constraint

**DOI:** 10.3390/life5021301

**Published:** 2015-04-24

**Authors:** Steven E. Massey

**Affiliations:** Biology Department, PO Box 23360, University of Puerto Rico—Rio Piedras, San Juan, PR 00931, USA; E-Mail: stevenEmassey@gmail.com; Tel.: +1-787-598-4859

**Keywords:** genetic code, codon reassignment, proteome size, proteomic constraint, genomic information content, neutral emergence, pseudaptation, Frozen Accident, DNA repair, mutation rate

## Abstract

The standard genetic code (SGC) is central to molecular biology and its origin and evolution is a fundamental problem in evolutionary biology, the elucidation of which promises to reveal much about the origins of life. In addition, we propose that study of its origin can also reveal some fundamental and generalizable insights into mechanisms of molecular evolution, utilizing concepts from complexity theory. The first is that beneficial traits may arise by non-adaptive processes, via a process of “neutral emergence”. The structure of the SGC is optimized for the property of error minimization, which reduces the deleterious impact of point mutations. Via simulation, it can be shown that genetic codes with error minimization superior to the SGC can emerge in a neutral fashion simply by a process of genetic code expansion via tRNA and aminoacyl-tRNA synthetase duplication, whereby similar amino acids are added to codons related to that of the parent amino acid. This process of neutral emergence has implications beyond that of the genetic code, as it suggests that not all beneficial traits have arisen by the direct action of natural selection; we term these “pseudaptations”, and discuss a range of potential examples. Secondly, consideration of genetic code deviations (codon reassignments) reveals that these are mostly associated with a reduction in proteome size. This code malleability implies the existence of a proteomic constraint on the genetic code, proportional to the size of the proteome (P), and that its reduction in size leads to an “unfreezing” of the codon – amino acid mapping that defines the genetic code, consistent with Crick’s Frozen Accident theory. The concept of a proteomic constraint may be extended to propose a general informational constraint on genetic fidelity, which may be used to explain variously, differences in mutation rates in genomes with differing proteome sizes, differences in DNA repair capacity and genome GC content between organisms, a selective pressure in the evolution of sexual reproduction, and differences in translational fidelity. Lastly, the utility of the concept of an informational constraint to other diverse fields of research is explored.

## 1. The Genetic Code: Near Optimal and Near Universal

“Theory space” has been well covered when it comes to the potential pathways that may have led to the present day standard genetic code (SGC) [1]. However, while it is largely accepted that the genetic code underwent expansion during its evolution, there is no consensus regarding the specific route that led its development, which may indeed be unknowable [2]. The reason why these considerations are important relates to a central concern in biology, that of “genotype—phenotype mapping”. This refers to the inference of phenotype from genotype, which can ultimately be reduced to the mapping of codons to amino acids, defined by the SGC. This means that the “onset of coding”, the transition to the protein world and the establishment of codon—amino acid mapping, is of key importance in evolutionary biology. While the exact details may never be known, we contend that universal and generalizable features and principles may still be elucidated. This type of “code breaking” may require a combination of evolutionary theory, simulation and experimental considerations, combined with a perspective that integrates more wide ranging fields of study. There are two key observations regarding the SGC that will be utilized in this work. Firstly, the genetic code is at least near optimal for the purpose of error minimization (the minimization of the deleterious impact of random point mutations), and secondly it is near universal. We propose that study of these two key observations can potentially reveal fundamental aspects of molecular and organismal evolution. In particular, we propose that ideas from complexity theory can explain these two features of the SGC. For this, the idea of the neutral emergence of mutational robustness will be used, emergence being a characteristic feature of complex systems, and the concept of genomic information content, which is a measure of organismal complexity.

The arrangement of codons to amino acids (*i.e.*, codon—amino acid mapping) in the SGC is such that the deleterious effects of point mutations are minimized, compared to randomly generated codes; this error minimization is a form of mutational robustness. Error minimization has been assumed to be the result of direct selection (known as the “physicochemical theory” [3,4,5,6]), however we will show that this is not necessarily the case. A debate has centered on the extent of the error minimization in the SGC, with some workers claiming that the code is “one in a million” [7] and optimal [8,9,10,11,12], with other studies showing that the code is “near optimal” [13] (though see [14]), [15,16,17,18,19,20,21]. An important caveat is that some analyses of code optimality depend on amino acid similarity matrices to measure error minimization that are inherently biased in that they rely on empirically observed amino acid substitutions. The relative frequency of these are affected by the structure of the genetic code itself, because amino acids encoded by codons that differ by one nucleotide will be substituted more frequently than those that differ by two nucleotides. This potentially produces inflated measures of genetic code optimality for the property of error minimization [22,23]. Therefore, in order to calculate error minimization, substitution derived amino acid similarity matrices should be avoided, and matrices based on physicochemical amino acid similarity are more appropriate, the first of which was derived specifically in order to examine genetic code evolution [24]. The exact degree of optimality of the SGC remains to be established, and varies depending on assumptions made, however the SGC is at least near optimal for error minimization. A more important question then becomes, “How did the error minimization property arise?”, which is the first of the two topics addressed in this paper.

The second key feature of the genetic code that will be explored here is that it is not universal, having undergone alterations in some genomes. When the genetic code was first elucidated in the 1960s, it was found to be identical in phylogenetically diverse lineages such as metazoa, fungi and bacteria, hence it was supposed to be universal, and a striking confirmation of Darwin’s radical idea of common descent. However, beginning with the sequencing of the human mitochondrial genome in the early 1980s [25], it was shown that there were some deviations from the genetic code, termed “codon reassignments” or “alternative” genetic codes. Now, a range of codon reassignments are known from a range of different genomes, however, there is no consensus as to the mechanism that has produced them, or their driving force. A central problem is that of Crick’s Frozen Accident [26], which asserts that the reason for the widespread distribution of the SGC (only a minority of genomes use deviant genetic codes), is that any changes to the genetic code would be catastrophic to the organism. This is because reassigning a codon to a new amino acid effectively involves mutating every single occurrence of that codon in the genome, which is expected to lead to a massive deleterious mutational load. Consistent with this, it is routinely observed in a range of organisms that mutation of a tRNA anticodon to recognize a non-cognate codon(s) is lethal, as is altering tRNA identity elements so that a tRNA is misrecognized by a non-cognate aminoacyl-tRNA synthetase and so charged by a non-cognate amino acid. However, a key observation is that codon reassignments are particularly common in certain genomes such as non-plant mitochondria and intracellular bacteria, suggesting the existence of predisposing factors. We propose that one key factor is reduced proteome size (P, the total number of codons/amino acids encoded by the genome) and explore how this may act as an evolutionary constraint on the genetic code. We then examine how genomic information content may act as an evolutionary constraint on other elements of the genetic information system responsible for fidelity, providing indirect evidence for its role in influencing genetic code malleability.

## 2. Neutral Emergence of Error Minimization in the Genetic Code

### 2.1. The Non-Adaptive Code Hypothesis

When biological systems show a high level of optimality, the default assumption is that these traits are the direct product of natural selection, which is an optimizing process. However, while the majority of phenotypic traits are undoubtedly directly selected for, it is not necessarily correct to assume that all phenotypic traits have been produced by the direct action of selection and have adaptive value, as pointed out by Gould and Lewontin [27]. These authors envisaged that there may exist some phenotypic traits that have not been produced by the direct action of selection and that lack adaptive value, *i.e.*, do not contribute to the fitness of the organism; these were termed “spandrels”. We go a step further and propose that there are some traits that have adaptive value (*i.e.*, increase fitness) that have *not* arisen by the direct action of natural selection, and that the genetic code may provide a prime example. We have termed such beneficial traits “pseudaptations” [21,28,29], given that there may be a tendency to erroneously describe them as true adaptations, which are fitness increasing traits that are directly selected for. The case of error minimization in the genetic code will be discussed first, as this may present the paradigm of a pseudaptation. Then, other potential pseudaptations will be identified in a range of systems, in order to identify commonalities with the error minimization of the SGC.

In order for an error minimized genetic code be directly selected for there needs to be a searching of code space, the space of alternative genetic codes, for an optimal, or near optimal, code [30]. Two widely discussed potential mechanisms for how codon reassignments occur are the codon capture mechanism [31,32] and the ambiguous intermediate mechanism [33,34]. The codon capture mechanism proposes a complete loss from the genome of the codon undergoing reassignment. This is proposed to occur via strong GC or AT mutation bias, which is expected to lead to extremes of genome GC or AT content, whereby AT rich or GC rich codons respectively are expected to disappear from the genome entirely. The disappearance of the codon avoids lethal disruption to the proteome, caused by altering the codon—amino acid mapping of the SGC. However, there are only a few examples of complete codon loss in present day organisms (*Mycoplasma capricolum* has lost the CGG codon from its genome [35], and *Micrococcus luteus* has lost AGA/AUA from its genome [36]), and variations in genome GC/AT content along the chromosome also means that complete codon loss is difficult [34]. This mechanism would have been more likely to occur in organisms with smaller values of P, where total codon loss from the genome is more likely [31]. Thus, for this mechanism, P is expected to exert a constraint on the efficacy of a reassignment. However, a strong argument against the occurrence of this mechanism of codon reassignment via GC/AT mutational pressure is that codon reassignments of AT rich codons are often observed in AT biased genomes [37], for example the UGA stop codon reassignment discussed below. AT rich codons are highly unlikely to have disappeared in AT rich genomes and so these observations are inconsistent with the codon capture mechanism.

The alternative ambiguous intermediate mechanism proposes that the reassigned codon did not disappear from the genome, but underwent a dual ambiguous stage where the codon had the original amino acid identity, and that of its new amino acid assignment. This process may either have been adaptive, which would imply that reassigning all codons simultaneously conferred a fitness benefit, or it was disruptive to the proteome, in which case the level of disruption would be less in smaller proteomes [37]. In the second scenario in particular, a smaller value of P is likely to facilitate the reassignment.

The “adaptive code hypothesis” (analogous to the physicochemical theory) proposes that error minimization has been directly selected for [8]; and initially this might appear a reasonable assumption. However, when one starts to probe potential mechanisms by which error minimization might be directly selected for, problems arise. Firstly, there has been a long standing discussion as to whether mutational robustness can be directly selected for [38]. Here, it is important to distinguish between intrinsic and extrinsic robustness [39]. Extrinsic robustness is externally imposed on a system and includes homeostatic mechanisms. This type of robustness can be directly selected for, such as the heat shock response, for example. Intrinsic robustness is an innate property of networks (deriving mostly from their topology) or sequences. Here, it is ambiguous if and when intrinsic robustness can be directly selected for, or whether it is a property that emerges as a side-product of selection for some other function (which would make it a pseudaptation; discussed in more detail below). The difficulty of selecting for an intrinsically robust sequence or network topology hinges on the argument that natural selection does not have foresight, and so it is difficult to select a robust sequence on the remote chance that it may experience a deleterious mutation several generations in the future. This implies that selection for robustness is difficult in low mutation rate regimes, and so it has more often been associated with high mutation rates [38]. Even here, direct selection is difficult, and when robustness in these regimes is produced it seems to be a second order effect [40]. While selecting for mutationally robust sequences may be difficult to envision mechanistically, a possible solution is to select at the transcriptional/translational level; this is more attractive given the high level of transcriptional/translational errors compared with the underlying genotypic mutation rate [41], and the occurrence of multiple transcripts, which means that within the lifetime of a cell, a particular site in a sequence is considerably more likely to undergo an error. With this in mind, there is evidence that transcriptional/translational selection alters synonymous codon usage in order to reduce the deleterious effects of transcriptional/translational errors [42,43,44,45,46], although one study finds no evidence of his type of error minimization [47]. Overall, it seems that transcriptional/translational error minimization is a weak selective force given that it is particularly associated with high expression level [43,44], location within the gene [40,41], and may be reduced by prevailing codon usage [48,49]. So, whether there was enough selective pressure to lead to error minimizing codon - amino acid mappings during the evolution of the SGC is debatable.

Secondly and more crucially, codon reassignments are necessary in order to search code space for an error minimized code. This is mechanistically difficult because the numbers of alternative codes is high, and because codon reassignments are disruptive (according to the ambiguous intermediate mechanism) or require extreme GC/AT bias (as in the codon capture mechanism). For a codon reassignment to be selected because of a resulting improvement to the overall error minimization of the code, the improvement would have to be large enough to outweigh overall proteome disruption, which seems unlikely [26,50]; this may be summarized as the question “Is the benefit from improved error minimization resulting from a codon reassignment greater than the cost of the proteome disruption?”. In addition, there is a scarcity of evidence that present day codon reassignments have led to an improvement in error minimization [8,51,52], or that they have any other adaptive value. Problematically, simulations show that codes get trapped in sub-optimal local minima when the codon capture mechanism is utilized to search code space for error minimizing genetic codes, given the intrinsic constraints of the mechanism [30]. This indicates that code optimization was unlikely to occur via this mechanism. Utilizing the ambiguous intermediate mechanism, numerous codon reassignments are required to produce a code with error minimization properties similar to the SGC (>20 under the specific model constraints [30]), and this does not incorporate unknown constraints imposed by the properties of ancestral aaRSs and tRNAs. Utilization of the ambiguous intermediate mechanism for code optimization would require therefore multiple ambiguous decoding phases.

So, if error minimization is difficult to accomplish via direct selection, how did it arise? A potential answer lies in Crick’s observation that genetic code expansion was likely facilitated by the duplication of adaptor molecules and charging enzymes, with the result that “similar amino acids would tend to have similar codons” [26,53]. Taken further, it can be shown that no matter which path genetic code expansion takes, if new amino acids are added to related codons of related amino acids (mimicking the process of adaptor and charging enzyme gene duplication, necessary for genetic code expansion), then a degree of error minimization arises, often equivalent or superior to that of the SGC [21]. Remarkably, under certain similarity criteria used to select which amino acids are added to the expanding code, 22% of alternative codes produced have error minimization superior to the SGC [21]. This process may have occurred via the gene duplication of tRNAs and proteinaceous aaRSs driven by the selective benefit of adding new amino acids to the genetic code, or of primordial adaptor molecules and charging enzymes, irrespective of their exact biochemical nature. Pertinently, it has been shown that RNAs can have aminoacylation properties [54,55], and other organic heteropolymers could also possess similar activities. A key problem in trying to infer genetic code expansion from the present day proteinaceous aaRSs is the classic chicken in the egg question. *i.e.*, if the genetic code was incomplete, how could proteinaceous aaRS be encoded in order to incorporate new amino acids into the expanding code [50]? It seems some allowance needs to be made for the incomplete amino acid complement encoded by an incomplete genetic code in studies that try to link the phylogenetic relationships of the present day aaRSs with their amino acid/tRNA specificities in order to make inferences about genetic code evolution, and so their conclusions should be treated with caution.

**Figure 1:**
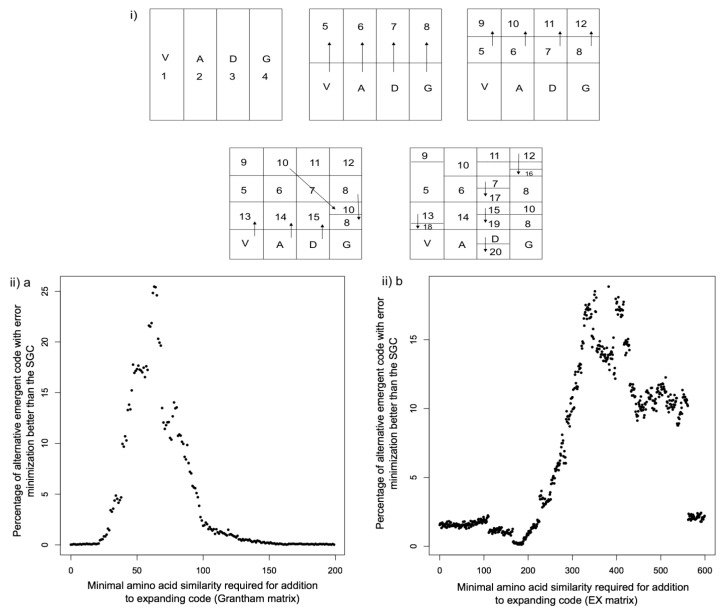

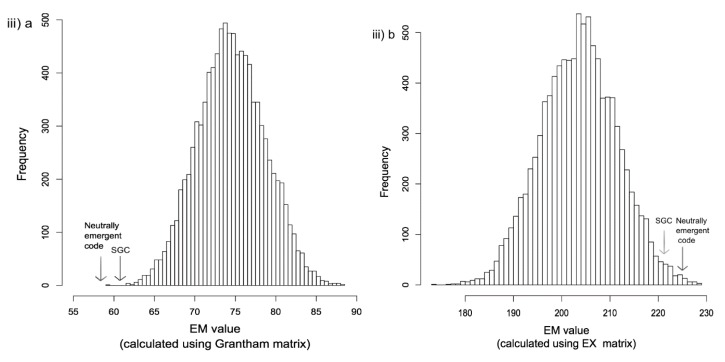
How error minimization may neutrally emerge in genetic codes.

When the Grantham physicochemical amino acid similarity matrix [56] is used to study the error minimizing efficiency of the SGC, a marked degree of optimality is observed, with the SGC better than 99.7% of randomly generated alternative genetic codes for the property [21] (and shown in Figure 1(iii)a). However, it has been pointed out that the Grantham matrix itself is biased by modification to fit the observed frequencies of amino acid substitutions [53]. A solution is to use matrices designed to avoid this type of bias, such as the Exchangeability (EX) matrix, which is derived from the experimentally determined effects of amino acid substitutions on protein activity [57]. When this matrix is utilized it can be shown that the SGC is better than 98.6% of randomly generated genetic codes (shown in Figure 1(iii)b), which is consistent with its near optimality. The two different matrices were used to explore how error minimization can arise without direct selection, utilizing a scheme consistent with a “213” mechanism of genetic code expansion, where the 2nd codon position acquires meaning first, followed by the 1st position, and lastly the 3rd position [58] (scheme illustrated in Figure 1(i)). A range of similarity thresholds was used, in order to choose which amino acid to add to the expanding code, based on similarity to the “parent” amino acid already present in the evolving code, illustrated in Figure 1(i). If no amino acid was available according to the similarity criteria, then a random amino acid was added. 10,000 codes were generated for each similarity threshold, and the proportion of codes with better error minimization than the SGC is shown in Figure 1(ii)a (Grantham matrix), and in Figure 1(ii)b (EX matrix). In the case of the Grantham matrix, for some similarity thresholds over 25% of codes were better than the SGC for error minimization. For the EX matrix, for some similarity thresholds over 18% of codes were better than the SGC. These results confirm that a stepwise process of genetic code expansion via gene duplication of adaptor molecules and charging enzymes is a viable explanation for the presence and degree of error minimization in the SGC.

A simple change to the rules for adding new amino acids to the expanding code produces striking results. When the unassigned amino acid that is most similar to the “parent” amino acid is added to the expanding code, according to the scheme in Figure 1(i), a superior genetic code to the SGC is produced when using both the Grantham matrix (Figure 1(iii)a), and the EX matrix (Figure 1(iii)b). These results confirm that direct selection is not necessary to produce an error minimized genetic code, but that it may “emerge” due to simple rules imposed during code expansion, *i.e.*, that similar amino acids are added to related codons of a related amino acid already present in the growing code. This rule of addition adheres to standard biochemical and molecular evolutionary principles; enzyme duplicates typically show substrate specificity related to the parent enzyme, and tRNA duplicates are likely to have related anticodons, given that homologous sequences are characterized by sequence similarity. This finding allows us to distinguish between different theories of genetic code evolution. For example, the data are inconsistent with the stereochemical theory [59,60,61] that proposes a direct interaction between codon/anticodon and amino acid is responsible for the structure of the genetic code. While this might still lead to similar amino acids being recognized by similar anticodons, it does not involve a process of code expansion via gene duplication. The data are also incompatible with the physicochemical theory, which proposes that direct selection is responsible for error minimization. Lastly, the coevolution theory proposes that the genetic code expanded via the addition of biosynthetically related amino acids [62,63]. If this expansion were to occur via gene duplication [64,65] then our results show how error minimization could have arisen concomitantly.

Two simulations were conducted in order to demonstrate how error minimization may neutrally emerge during genetic code expansion. The Grantham matrix (a) and the Exchangeability matrix (b) were utilized to measure amino acid similarity. The Grantham matrix is derived from physicochemical considerations, while the EX matrix is derived from fitness effects on proteins resulting from amino acid substitutions. In order to measure error minimization, the Error Minimization (EM) value was calculated as described previously [21]; this is the average amino acid similarity resulting from a single point mutation for a given genetic code. The EM value is matrix dependent; in the case of the Grantham matrix the smaller the EM value, the greater the level of error minimization, and in the case of the EX matrix, the larger the EM value, the greater the level of error minimization. The simulation was conducted as follows. A scheme consistent with the “213” mechanism of genetic code expansion [58] was utilized:

(i) (reproduced from [21]). This involves the initial assignment of V, A, D and G to the 2nd codon position nucleotide T, C, A and G, respectively, which reflects the SGC. Then the 1st codon position nucleotide acquires meaning, followed by the 3rd codon position nucleotide. Amino acids were added to the expanding genetic code, following the illustrated scheme, according to two different criteria:

(ii) amino acids were added to the expanding code if they were below a similarity threshold relative to the amino acid of the “parent” codon. 10,000 iterations were conducted for each threshold, and the percentage of genetic codes with EM values superior to the SGC are displayed. For the Grantham matrix, smaller values indicate greater amino acid similarity, while for the EX matrix larger values indicate greater similarity.

(iii) the most similar amino acid to the amino acid of the “parent” codon out of all unassigned amino acids was added to the expanding code. Only one iteration was conducted as there is only one pathway of code expansion that can be followed, for each matrix. The EM value of the “neutrally emergent code” thus produced was compared to that of the SGC, and to 10,000 randomly generated codes.

“Emergence” refers to novel patterns and properties in a complex system arising from the interaction of substituent subcomponents, or “the whole is greater than the sum of the parts”. Simple rules of interaction are able to produce emergent properties, reflected in the code simulations described. Emergence is observed in a wide range of disciplines and fields of study, and life itself can be viewed as an emergent property [66]. Crucially, the emergence of error minimization in genetic code evolution occurs no matter what pathway of genetic code expansion is followed [21], and so the historical route to the SGC does not influence our main conclusion that at least a portion of the error minimization has arisen without the direct action of natural selection. In addition, we present conditions under which codes with superior EM properties to the SGC can easily arise. Because we show that error minimization is an emergent property that is not directly selected for, we term this process *neutral emergence*, in contrast to better known biological examples of *selected emergence*. These two forms of biological emergence will be discussed in more detail next.

### 2.2. Emergence in Biological Systems

The genetic code evolution simulations described demonstrate that the error minimization of the SGC may be an emergent property not directly selected for, and so this prompts a search for analogous properties in biological systems. The question we ask is whether the case of the SGC is a unique scenario or whether this process of neutral emergence is found elsewhere. There is a long list of examples of selected emergence in biological systems, at many different levels of organization. These include swarm intelligence (e.g., bees foraging), metabolic flux, fractal geometry in circulatory structures (a way to improve efficiency, [67]), the action potential of neurons, self assembling crystal-like structures (e.g., in virus capsids), and potentially consciousness [68]. Emergent properties are features of complex systems, and in non-biological systems these are typically not directly selected for, but are passively emergent. This is analogous to the process of neutral emergence that we have identified as potentially operating during the evolution of the SGC. This implies that we may expect similarities between emergent properties in non-biological systems and neutrally emergent properties in biological systems.

**Figure 2:**
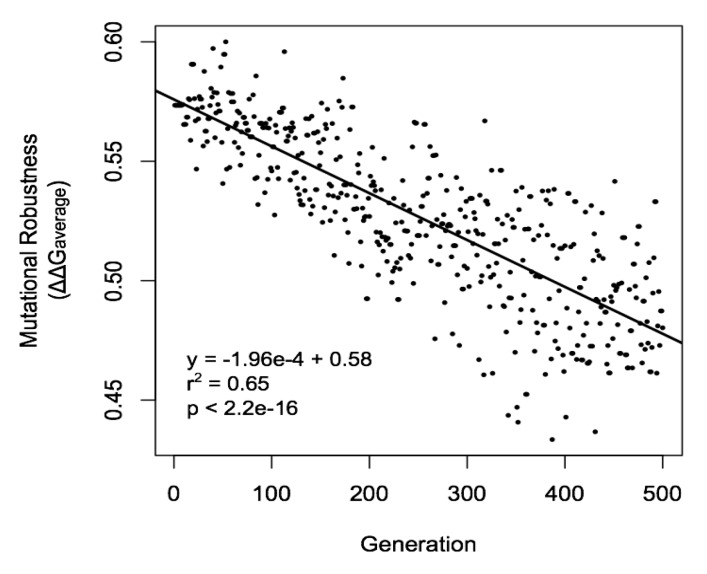
Neutral emergence of mutational robustness in a population of proteins.

Neutrally emergent properties in biological systems may potentially be beneficial, or of no fitness benefit. A major category of beneficial traits that may be neutrally emergent are associated with robustness; this includes the error minimization of the SGC, which is a form of mutational robustness. These are listed in Table 1a. In an interesting parallel, many non biological complex systems also show the property of robustness, often associated with network topology [69], an emergent property [70]. In Figure 2, we show another example of how mutational robustness may neutrally emerge, this time within protein structures. In this example, a population of protein structures is subject to negative selection for structural stability. Over time, on average the structure becomes more mutationally robust, even though this property is not being directly selected for. This observation may be explained in the framework of neutral network theory [71,72]. This proposes that a protein or RNA sequence drifts through a sequence space of neutrally connected sequences, which avoids disruption to the structure; this is termed a “neutral network”. This movement happens stochastically until the sequences reaches a more highly neutrally connected region of the network; the sequences are likely to be move to these regions simply by chance. Here, the sequence is more robust as a greater proportion of potential mutations it may undergo are neutral, given that the region of the network has a higher proportion of neutral connections [73,74,75]. In the simulation, mutations that do not affect stability are classified as effectively neutral and may stochastically spread through the population, and so the protein sequences will change neutrally until they enter a more highly connected area of sequence space, which confers higher mutational robustness. This is an example of how intrinsic sequence robustness may evolve via non-adaptive processes, and provides an additional example of neutral emergence of mutational robustness to that of error minimization in the SGC.

A population of single domain proteins of identical structure, utilizing the 3D structure of leech eglin C (1EGL.pdb), was subject to negative selection for stability in a Wright-Fisher evolutionary simulation. A population size of 1000, mutation rate of 0.005 per nucleotide per generation, and a transition/transversion ratio of 2 were utilized. The fitness function was the effective free energy of folding (ΔG_f_), calculated from the amino acid sequence of each protein in the population threaded onto the starting 3D structure, using a coarse grained contact model whereby each amino acid was represented by a coarse grained site centered on the 1^st^ carbon atom of the amino acid functional group [76]. ΔG_f_ was −21.05 for the starting sequence VSLNVITPLCTRVEKCIQIPTVVAVLRAAAVIVWIGILQSPVYGLVLRLALYDYALGRLGSLNQAVYVPL, which was a randomly generated sequence chosen for its low mutational robustness, defined as the average ΔΔG resulting from a point mutation of the gene sequence encoding the protein (ΔΔG_average_). A selection threshold of 0.29 above and below the starting value of ΔG_f_ was utilized; for each generation, sequences that had ΔG_f_ values outside these thresholds as the result of mutations were removed from the population. ΔΔG_average_ of the most common member of each generation was calculated and plotted. The methodology is described in more detail in [29].

A second category of traits that may be neutrally emergent are associated with evolvability (Table 1b). There is an ongoing debate regarding the factors that promote evolvability. Foresight is required to directly select evolvability, given that improved evolvability implies future rather than immediate benefits to the organism, and this is problematic from a theoretical point of view [77]. Table 1b lists the potential driving forces behind a series of biological processes, that may indirectly result in increased evolvability, in many, but not all, cases a process of neutral emergence may be responsible (indicated on Table 1a,b). Error minimization of the SGC has been proposed to increase adaptability [78,79], and this illustrates the close connection between robustness and evolvability [80,81]. Increased mutational robustness may allow a more efficient exploration of sequence space and novel evolutionary solutions, however at the cost of decreased phenotypic variation which is expected to reduce the strength of selection. Thus, error minimization in the SGC may be beneficial in two regards; it reduces the deleterious impact of mutations and may increase evolvability. A third category of emergent property that is not listed in Table 1a,b is that of intrinsic noise. Intrinsic noise refers to noise that is inherent within a system. The occurrence and role of intrinsic noise is an exciting topic in biology, and its evolutionary implications are just beginning to be explored [82]. The extent to which this type of emergent property is beneficial, how often it is directly selected for, or if it is neutrally emergent, remains to be determined.

Table 1Potential pseudaptations in biological systems. Beneficial traits that may have arisen as a side effect of selection for a different trait (“pseudaptations”) are listed and comprise two main categories: (**a**) robustness related; (**b**) evolvability related. These traits are often proposed to have arisen by the direct action of natural selection, but evidence is listed here that they have arisen as fortuitious “side-products” of selection for a different trait. Those traits proposed to have arisen by a process of neutral emergence are indicated by *.

**(a):** Robustness related potential pseudaptations.

Trait	Potential driving force	Indirect benefit (neutrally emergent*)
Increased proteome hydrophobicity in AT rich genomes	Hypothesized that AT bias may arise neutrally via changes in mutation bias [83], one cause of which may be loss of DNA repair genes [84], which may indirectly be a result of a reduction in P [85,86,87], and this work. AT rich codons encode more hydrophobic amino acids, so AT bias results in more hydrophobic proteins	Increased hydrophobicity of proteome results in increased protein folding stability [88] *
Scale free structure of metabolic networks	There is evidence preferential attachment has given rise to the scale free property [89]	Robustness to gene deletion [90,91] *
Scale free structure of protein interaction networks	There is evidence that preferential attachment has given rise to the scale free property [92,93]	Robustness to gene deletion [94,95] *
Scale free structure of gene regulatory networks	There is evidence a combination of gene duplication and preferential attachment are responsible for the scale free property [96]	Robustness to mutation [97] *
Survival of the flattest	Survival of the flattest refers to the increase in number of robust organisms in a population when mutation rates are high. This neutrally emerges in digital organisms [98] and RNA viruses [40] in the absence of direct selection for the property	Increased robustness of the population to mutation *
Mutational robustness of protein and RNA structures	Mutational robustness in RNA secondary structures [74,99], protein 2D lattices [73,100] and 3D coarse grained protein models [29] neutrally emerges via random movement on a neutral network as a result of genetic drift	Increased structural robustness to mutation *
Error minimization of the genetic code	There is evidence that error minimization neutrally emerged during genetic code expansion via gene duplication of adaptor molecules and charging enzymes [21] and this work.	Error minimization reduces the deleterious impact of point mutations, transcriptional and translational errors *
Genetic dominance	It has been proposed that genetic dominance is selected for to increase metabolic flux [101], or that it is a side product of enzyme kinetics [102]	Increased mutational robustness [38]
Enhanced DNA repair in Deinococcus radiodurans	The ability to withstand dessication may have led to the enhanced repair of double stranded breaks [103]	Enhanced repair of double stranded breaks also leads to radiation resistance in this species. Radiation is rarely encountered in nature, so it is unlikely radiation resistance was directly selected for [103]

**(b):** Evolvability related potential pseudaptations.

Trait	Potential driving force	Indirect benefit (neutrally emergent *)
Sexual reproduction	The purpose of sexual reproduction has been proposed to be DNA repair via recombination [104]	Recombination leads to a reduction in the Hill-Robertson effect, enhancing the strength of selection *
Segmentation of virus genomes	The role of virus genome segmentation has been linked to differential gene expression [105]	In cystoviruses, segmentation leads to random assortment, and subsequent amelioration of linkage disequilibrium [106], increasing the power of selection. Likewise, in the influenza virus segmentation may increase the strength of selection [107] *
Protein domain shuffling	Domain shuffling is facilitated by the occurrence of introns [108], which have a variety of functions, however the role of most of them remains to be established [109]	Domain shuffling has been linked to evolutionary innovation [110] *
Reduced population size	Many factors may act to reduce population size and it is unlikely to be directly selected for	Ability to traverse evolutionary barriers [111,112] *
Nonfunctional DNA in higher eukaryotes	The function of the majority of intron sequences and intergenic DNA, if any, has not been established. Notably, overall there is a lack of sequence conservation, indicating a lack of sequence specific selection [113]	Longer introns and intergenic DNA regions lead to an increase in recombination events, reducing the Hill-Roberston effect and so increasing the strength of selection [114,115,116] *
Evolutionary capacitance of HSP90	HSP90 is a normal part of the stress response in the eukaryotes	HSP90 acts to store cryptic genetic variation, this is exposed in times of stress due to a reduction in the concentration of free HSP90 [117,118] *
Evolutionary capacitance of complex gene regulatory networks	Gene regulatory network structure is driven by the addition and removal of nodes, according to the immediate selective benefit	The loss of a gene enhances the phenotypic variation of remaining components of the network, and this promotes evolvability, this effect is not dependent on network topology [119] *
Error minimization in the SGC	There is evidence that error minimization has neutrally emerged as a consequence of genetic code expansion over time [21,30], and this work	Error minimization has been proposed to result in the increased evolvability of proteins [78,79] *
Elevated mutation rates in RNA viruses	The ultimate cause of elevated mutation rates in RNA viruses has not established, but reduced P may be a factor [85] and this work. The proximate cause of the elevated mutation rates is a lack of proofreading in the replicative polymerase	Elevated mutation rates increase the ability to evade the host immune system and adapt to drug treatments
Ambiguous decoding of the CUG codon as both serine and leucine in *Candida* yeasts	The ambiguous decoding of CUG [120] appears to have been a factor in the codon reassignment of CUG leu→ser [120]	Ambiguous CUG decoding produces elevated levels of HSPs and this enhances survivability in challenging environments [121]
Polyploidy	Polyploidy is caused by abnormal cell division	Polyploidy is proposed to result in increased evolvability in plants [122,123]
Lateral gene transfer (LGT) in prokaryotes	LGT may have a role in DNA repair of the prokaryotic genome [124] or may be a side-product of the uptake of DNA as carbon and energy source [125]	LGT leads to increased evolvability in response to environmental challenges

### 2.3. Pseudaptations: Beneficial Traits that Have not Been Directly Selected for

There is an increasing number of examples of potential pseudaptations, beneficial traits that have not been directly selected for, which includes the error minimization property of the SGC. We list these in Tables 1 and this allows us to identify two main categories of pseudaptations; robustness related and evolvability related. Most of both the robustness and evolvability related traits appear to arise by neutral emergence (indicated). Thus, we are able to identify two major exceptions to the tautology that all adaptations, *i.e.*, fitness increasing traits are the product of natural selection, and defined as such; that of robustness and evolvability. This leads us to propose that adaptations are better defined as beneficial traits directly selected for, that increase the fitness of the organism, while pseudaptations are beneficial traits that increase the fitness of the organism, which however have not been directly selected for that purpose.

## 3. Proteome Size as a Constraint on the Genetic Code

### 3.1. Unfreezing of the Code

Crick’s Frozen Accident theory proposes that there was a stage when the genetic code was fluid, and that it “froze” when the numbers of proteins (and so its proteome size) in the ancestral lineage increased, which resulted in an increased constraint on the code [26]. We have termed this as a “proteomic constraint” on the genetic code [37,85], and this concept proposes that genetic code changes result in a larger mutational load in larger proteomes. The idea of “freezing” implies that if P reduces in size then the code can be “unfrozen” and therefore malleable. This relationship between P and genetic code malleability can be shown via simulation, with codon reassignments occurring more frequently when P is smaller [126]. Consistent with these considerations, when the relationship between P and numbers of codon reassignments in mitochondria was examined [37], it was found that the number of mitochondrial codon reassignments is positively correlated with mitochondrial proteome size. More recently, a number of additional codon reassignments have been discovered in bacteria, listed in Table 2. Strikingly, all bacterial codon reassignments are found in bacteria with small values of P, and remarkably, the identical codon reassignment (UGA stop→trp) has evolved several times independently. The frequency of the UGA→trp reassignment in multiple systems has been attributed to the widespread naturally occurring UGA read through activity of trp-tRNA in a range of translation systems [127,128] (and see [129] for a mutant trp-tRNA with readthrough activity with a standard anticodon), and is a potential example of an evolutionary predisposition or “preadaptation” [37]. The association of the codon reassignments in Table 2 with reduced values of P, and the independent occurrence of identical codon reassignments, is strongly reminiscent of mitochondrial codon reassignments and implies a common codon reassignment mechanism and driving force in these two different types of genomes. In addition, the bacteria in Table 2 mostly have strong genome AT biases, again in common with mitochondria. This observation may indicate a link between loss of DNA repair and reduced P, discussed in the next section. A commonality between mitochondria and the bacteria in Table 2 is their intracellular habitat; mitochondria are intracellular organelles and the majority of the bacteria listed in Table 2 are intracellular. The intracellular lifestyle leads to a marked reduction in P, largely attributable to the loss of genes redundant with host functions, and genes no longer needed in an environment that varies little [130].

**Table 2:** Bacteria that have undergone codon reassignments.

Lineage and phylogenetic affiliation	Genetic code change	Genome size	Genome GC content	Elevated substitution rate?	Loss of DNA repair?	Habitat
Mycoplasmas (Mollicutes)	UGA (stop)→trp [131]	580–1359 kbp (Genbank)	25%–40% (Genbank)	Yes [132]	Yes [133]	Vertebrate cells
Spiroplasmas (Mollicutes)	UGA (stop)→trp [134]	940–2220 kbp [135]	29% [ 136]	Yes [132]	Yes [137]	Insect and plant cells
Ureaplasmas (Mollicutes)	UGA (stop)→trp [138]	750–950 kbp [139]	25% [139]	Yes [132]	Not determined	Vertebrate cells
SR1 bacteria (related to Chloroflexi)	UGA (stop)→gly [140]	1178 kbp [141]	31% [141]	Yes [141]	Not determined	Human body (extracellular), sediments
*Nasuia deltocephalinicol* (β proteobacteria)	UGA (stop)→trp [142]	112 kbp [142]	17% [142]	Yes [142]	Yes [142]	Circada (insect) cells
*Sulcia muelleri* (Bacteroidetes)	UGA (stop)→trp [142]	190 kbp [142]	24% [142]	Yes [143]	Yes [143]	Sharpshooter (insect) cells
*Hodgkinia cicadicola* (α proteobacteria)	UGA→trp [144]	144 kbp [144]	58% [144]	Yes [144]	Yes [144]	Circada (insect) cells

Codon reassignments are also occasionally found in systems other than mitochondria and bacteria. An AUA ile→met codon reassignment has occurred in the plastid *Lepidodinium chlorophorum* [145], which as an organelle likely possesses a reduced value of P. The *Candida* yeasts (CUG leu→ser [146,147], genome size ~15 Mbp), the *Spironucleus, Trepomonas and Hexamita* clade of diplomonads (UAA/UAG→gln [148,149] *Spironucleus* genome size ~12–18 Mbp) have smaller values of P. The oxymonads have undergone a UAA/UAG→gln codon reassignment [150], but their genome sizes remain to be determined. In contrast, the ciliates (UAA/UAG → gln in *Tetrahymena* and *Paramecium* [151,152], UGA stop→cys in *Euplotes* [153] UGA→trp in *Blepharisma* and *Colpoda* [154]) and Dasycladales/Cladophorales green macroalgae (UAA/UAG→gln [155,156,157,158] do not have small genome sizes and values of P, so a small value of P does not seem to be a universal facilitating factor for codon reassignments. However, reduced P is associated with the majority of codon reassignments in a variety of systems. This provides an intuitive explanation for code “unfreezing” resulting from a reduced constraint on codon–amino acid mapping, implying that P imposes a constraint on genetic code malleability; the so-called “proteomic constraint”.

### 3.2. Genomic Information Content as a Constraint on Genetic Fidelity

Given that accurate codon–amino acid mapping, resulting from accurate codon-anticodon and tRNA-aaRS recognition, is a form of genetic fidelity, then if P exerts a selective pressure on this fidelity, then it might be expected to influence other forms of genetic fidelity. In other words, given that codon–amino acid mapping is a form of genotype–phenotype mapping, the idea of a proteomic constraint should be extensible to other features of the genetic information system responsible for the fidelity of genotype–phenotype mapping. A range of examples of how genetic fidelity may be subject to the proteomic constraint has been examined [85], but an updated discussion will follow. Firstly, it should be noted that P is an approximation to the information content of a genome (I). While P is expected to exert a constraint on codon reassignments, other types of genetic information in the genome may have an influence on other forms of genetic fidelity and this genetic information may be identified by sequence conservation. For example, while the quantity of noncoding RNA may be more accurately calculated in the future in a wider range of organisms, due to improvements in prediction methods, it appears to constitute a significant proportion of the genome in humans, with 27 Mbp of predicted long noncoding RNAs in the human genome (Gencode release 21 [159]), compared with 43 Mbp of coding sequence (calculated using Augustus [160]). Promoter regions also contain sequence specific information, but unfortunately these are difficult to predict either from first principles, or using sequence conservation. Additional measures from the genome sequence might be incorporated for a more complete quantification of organismal complexity and genomic information content, I (discussed further below).

Given the idea that I may exert a selection pressure on genetic fidelity, proportional to its size, then a number of predictions may be made and also explanations for long standing evolutionary problems. Exploration of these may provide further support for the role of P and I in promoting genetic fidelity, and so may provide additional indirect evidence for the role of P in explaining the occurrence of codon reassignments, and ultimately help to understand the evolution of the SGC.

#### 3.2.1. Differences in Underlying Mutation Rates

**Figure 3:**
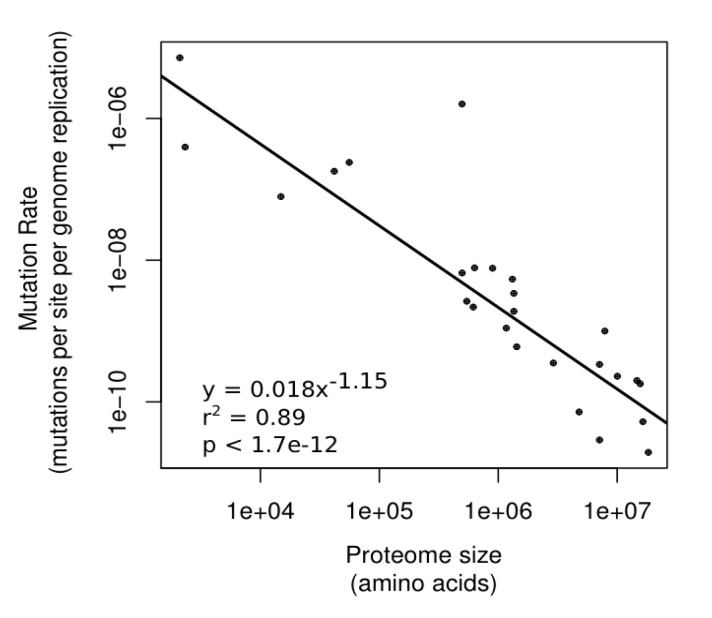
Relationship between proteome size and underlying mutation rates in diverse genomes.

A simple and intuitive idea is that the larger the amount of information in the genome, then the larger the mutational target. Given that most mutations are deleterious, this means there is a greater fitness cost to an organism with more genetic information, resulting from an increased mutational load. There is a general selective pressure to minimize the occurrence and effects of mutations, reflected in the diverse range of mutation avoidance, DNA repair and proofreading, and buffering mechanisms within the cell. So, it follows that genomes with larger amounts of genetic information should have a stronger selective pressure to evolve and maintain DNA repair and proofreading mechanisms, as they experience a higher mutational load. The mutational load is directly proportional to the length of the proteome in terms of amino acids (P); this means that the selective pressure to reduce mutations should also be directly proportional to P. This is expected to lead to an inverse relationship between the occurrence of mutations (expressed as the mutation rate, μ), and P. Thus, the relationship between μ and P can be related as follows:

(1) *μ* = *CP*^−1^ [85,161]

where C is termed the “Constraint factor”. C may vary according to each genome, and incorporates the genome wide strength of selection, which may be influenced by the genome’s effective population size (2N_e_ for a diploid population), and the average fitness effect of a mutation (which will be negative overall, as most mutations are deleterious). This average fitness effect can be expressed as its average selection coefficient, $\bar{s}$ , and may be influenced by recombination rate, given that increased recombination increases the strength of selection [162] In addition, the total number of fitness affecting mutations present (mutational load) is a factor, and is proportional to the product of P and heterozygosity per base pair (π), πP. The higher the mutational load, the greater the selective pressure to minimize μ, and so these are inversely related. Thus, for a diploid population the equation can be expressed as follows:

(2) $$\mu =k\left(2N_{e}\bar{s}\right){\left(\pi P\right)}^{-1}$$

where k is a proportionality constant. Importantly, the empirical data is consistent with a reciprocal relationship between μ and P in a wide range of eukaryotes, bacteria and DNA viruses (y = 0.018 x^−1.15^, r^2^ = 0.89, *p* < 1.7e^−12^, Figure 3), and indicates that P is the major determinant of mutation rates across genomes, explaining 89% of the variation in μ. This analysis is an extension the observed inverse relationship between μ and genome size in a range of microbes [163]. While N_e_ has been proposed to be the main determinant of mutation rates [164,165], this perspective does not take into account the expectation that N_e_ and $\bar{s}$ are inversely related to each other, with more deleterious mutations being present in organisms with smaller values of N_e_ [166]. This means that any effect from a reduced N_e_ will be counteracted by an increase in $\bar{s}$, neutralizing the influence of N_e_ on mutation rates.

The inverse relationship between µ and P implies that there is an increased selection pressure for DNA repair in organisms with larger values of P; this is because their lower values of µ implies more efficient DNA repair. This is indeed observed with mismatch repair genes and base excision repair genes [86], and for recombination repair genes [87], with these DNA repair genes more commonly found in bacteria with larger values of P. The relationship between P and DNA repair is discussed further below. The idea of a proteomic constraint also predicts that there should be a greater selection pressure for proofreading associated with larger values of P, and *vice versa* a reduced selection pressure associated with smaller values of P. While the lack of proofreading in RNA viruses, which have very small values of P, is consistent with this reduced selection pressure, in comparison with DNA based genomes they show an elevation in mutation rate more than simply their reduced values of P would suggest [85].

Two examples of acquisition and loss of proofreading in viruses, associated with changes in P, are consistent with operation of a proteomic constraint on replicational fidelity. Firstly, the nidoviruses are the largest RNA viruses; they have undergone a genome expansion (up to 32 kbp in size), with a concomitant increase in P. Accompanying this expansion has been the acquisition of RNA polymerase proofreading activity [167], consistent with the hypothesis that increased values of P increase the selective pressure to evolve proofreading. Secondly, the phaeoviruses are nucleocytoplasmic large DNA viruses (NCLDVs) that have undergone a recent reduction in genome size and concomitant reduction in P compared to the other NCLDVs, which have enormous genome sizes (phaeovirus genome sizes are 180–360 kbp [168]). This group of viruses has lost DNA polymerase proofreading activity [168], consistent with a reduction in the proteomic constraint on replicative fidelity. Eigen proposed that μ acts to restrict the information content of virus genomes [2,169], implying that these are close to an error threshold. This is opposite to the prediction of a proteomic constraint, which proposes that information content acts to restrict μ. A central problem with the Eigen hypothesis is that it requires foresight in order to evolve improved DNA repair and proofreading mechanisms before virus genomes can expand over time, and natural selection does not have foresight.

Equation (2) may be generalized to account for the total information content in the genome, *I*, as follows:

(3) $$\mu =k_{I}\left(2N_{e}\bar{s}\right){\left(\pi I\right)}^{-1}$$

where *I* is the number of nucleotides under selective constraint in the genome, and k_I_ is the associated proportionality constant. Negative selection acting on a nucleotide leads to conservation and indicates that it possesses information useful to the organism; the higher the level of conservation, the greater the informational value, and so it can be observed that it is the process of selection itself creates genetic information, as pointed out by Eigen [170].

#### 3.2.2. Loss of DNA Repair Genes and Changes in Genome GC Content

One of the great puzzles of genome biology is the wide variation in genome GC content in different organisms and organelles, first noted by Sueoka in bacteria in the 1960s [83]. He predicted that the variation may be due to differences in underlying mutational biases due to differences in DNA repair mechanisms, rather than the direct action of selection, an early premonition of the neutral theory of molecular evolution. More recently, the elevated AT content of genomes of reduced size such as organelles and intracellular bacteria (for example, see Table 2) has been attributed to the loss of DNA repair which characterizes these systems [85], however, the reason for this loss has been elusive. A reduction in N_e_ has been proposed as a cause [171], although it seems improbable that population effects could have such radical effects on gene complements. This is reflected in the empirical data whereby metazoa vary widely in their population densities [172], but vary little in their values of P. Examples from free living bacteria with reduced values of P are informative. The ocean bacteria *Prochlorococcus* and *Pelagibacter ubique* have undergone a reduction in P, and this has been accompanied by the loss of DNA repair [173], as have the free living SR1 group of bacteria (see Table 2). However, reduced N_e_ is not a good explanation for loss of DNA repair in these species, given that they likely possess substantial population sizes given their free living nature. For example *Prochlorococcus* has a very large value of N_e_, possibly the largest of any organism on the planet (~1.5 × 10^9^ [174]). An alternative explanation for the loss of DNA repair in these species is provided by the proteomic constraint hypothesis, which proposes that given their reduction in size of P there is less selective pressure to maintain DNA repair; this relationship is indeed observed in analyses of large numbers of free living bacterial genomes, where population effects are likely to be minimal [86,87].

The underlying mutation rates (μ) of a range of DNA virus, prokaryote and eukaryote genomes, obtained directly from the literature, were plotted against the respective proteome sizes (P), calculated by summing the numbers of codon present in all annotated ORFs from each genome (Supplementary Table S1). A correction was made for the number of germline cell divisions undergone by multicellular animals, as described in [85,161]; the resulting value of μ is per genome replication.

Likewise, the idea of a proteomic constraint can also explain the elevated substitution rates that accompany reductions in genome size, as is observed in intracellular bacteria and organelles. These elevations in substitution rate likely result from the loss of DNA repair and a subsequent elevation of μ, the underlying mutation rate, consistent with the inverse relationship shown in Figure 3 between μ and P. Consistent with the proposed influence of P on both the occurrence of codon reassignments and μ, all the bacterial lineages in Table 2 that have undergone codon reassignments also show elevated substitution rates and crucially, most have experienced a loss of DNA repair genes. Thus, in these examples a reduced value of P is associated with both a loss of the codon–amino acid mapping fidelity of the SGC, manifested by codon reassignment, and with a loss of replicative fidelity (which also disrupts genotype–phenotype mapping), manifested by loss of DNA repair and an elevation in substitution rates.

#### 3.2.3. The Evolution of Sexual Reproduction

Understanding the driving force behind the evolution and maintenance of sexual reproduction has been difficult. There are two major schools of thought; that it represents a form of DNA repair that operates via recombination repair mechanisms [104], or that it represents an adaptation to improve evolvability by more effectively combining beneficial mutations [175]. While it is little disputed that an effect of recombination is to increase the strength of selection [162], whether this enhanced selection itself is directly selected for, or simply a side-product of recombination has been extensively debated.

In addition, as discussed it is unclear if evolvability itself can ever be directly selected for [77]. In our view, the DNA repair hypothesis for the evolution of sexual reproduction is consistent with the observation that sexual reproduction evolved in eukaryotes, which typically have values of P larger than prokaryotes. Thus, we propose that the increased mutational load associated with an increase in mutational target resulting from an increase in information content in eukaryotes may have provided the selective pressure to evolve meiosis and concomitant improved DNA repair. Consistent with this, the presence of recombination repair genes is positively correlated with P in bacteria [87], and so a similar evolutionary force to evolve recombination repair in eukaryotes with larger values of P is reasonable. Given this scenario, improved evolvability resulting from an increase in recombination is a fortuitous side product, and so may represent a pseudaptation (Table1b). Importantly, recombination rate is negatively correlated with P in eukaryotes [85]. Recombination repairs double stranded breaks, which implies that larger proteomes have mechanisms to reduce the frequency of double stranded breaks, consistent with the proteomic constraint hypothesis, *i.e.*, there is an increased selective pressure to reduce the occurrence of these errors.

#### 3.2.4. Inefficient Organelle Protein Translation

Organelle rRNAs and tRNAs are marked by decreased stability, structural degeneration and functional inefficiency. Extreme cases are observed in metazoan mitochondria, where rRNA and tRNA sizes are extremely reduced and their secondary structures, which are highly conserved in other domains of life, are severely disrupted [176,177,178]. The accumulation of slightly deleterious destabilizing substitutions in organelle tRNAs and rRNAs has been viewed as an example of Muller’s ratchet [179,180,181], which proposes that deleterious mutations accumulate when recombination is reduced, resulting from the Hill-Robertson effect [182,183], which predicts a decrease in the strength of selection when recombination is reduced, as is the case in asexual organelles which do not undergo recombination. However, this explanation does not clarify why proteins encoded by the mitochondrion, such as cytochrome oxidase I, appear unaffected by deleterious mutations accumulation, and under “normal” evolutionary constraint. An explanation can be provided by the reduced value of P of the mitochondrion and a subsequent reduction in selection to maintain translational fidelity; this would be expected to affect components of the mitochondrial translation system that are involved in maintaining translational fidelity, but not the protein coding genes themselves, which are subject to selection at the level of the host. This is because translation is a form of genotype–phenotype mapping, and so the fidelity of this mapping is expected to be proportional to P; the reduced values of P in organelle genomes have potentially resulted in a reduced selection pressure to maintain translational fidelity.

### 3.3. Information as a Constraint in Diverse Systems

We have seen how genomic information content may act as a constraint on a range of error prone molecular processes, in that it exerts a cost via increased error load, and so it is interesting to compare it to other biological constraints. In a classic example, Haldane recognized that body size was subject to both physical constraints and also biological “design” constraints [184]. Insects illustrate this well; their body size is restricted due both to the limitations of gaseous diffusion, a physical constraint, and the arrangement of their circulation system, a design constraint. Genome information content can uniquely perhaps be classified as both a biological constraint, as it is created by natural selection, but also a physical constraint, in that it can be mathematically defined [185]. The latter is important because it means that information can be abstracted, and so we may expect to see analogies elsewhere in non-biological systems. In The Republic, Plato used the analogy of shadows on a cave wall, that were imperfect representations of the universal forms that generated the shadows. Platonic forms are thus universal concepts and mathematical truths that see an imperfect reflection in nature. As information may be described mathematically, it also constitutes a universal form. We might then expect that if information acts as a constraint and cost in biological systems, then parallels might be observed elsewhere in other complex systems, providing further indirect support for its role in genetic fidelity. Firstly, the information content and complexity of a system are positively related to each other. Complexity is difficult to define [186], and while Kolmogorov (algorithmic) complexity (which proposes that size of the simplest algorithm that may describe it is a measure of its complexity [187,188,189]) provides a universal definition, this measure is difficult to apply to organisms. However, genomic information content appears to be correlated with organismal complexity [190]. Thus, at the genomic level one measure of complexity is the amount of sequence specific information, I, which approximates to P. That P is an imperfect representation of complexity is clear from a consideration of the metazoa where P does not vary much, but complexity clearly does. This may be illustrated by comparing invertebrates with vertebrates; while the latter are more complex in terms of behavior, number of cell types, physiology, body sizes and brain structure, this is not reflected in a substantial increase in their value of P compared to invertebrates. An answer for this may lie in differences in the level and sophistication of alternative splicing and gene regulation in vertebrates. This may be partly measured by the quantity of noncoding RNAs, numbers of introns, numbers of transcription factors, and size of promoter regions, however at present these cannot be quantified with precision in non-model organisms. With these considerations in mind, an attempt to measure “effective genome information” has incorporated the factor of cell differentiation in multicellular eukaryotes in addition to P [190].

While it is not difficult to see that the increased complexity of a system, reflected in increased information content, leads to a greater chance of system failure, we know of no study that has compared complexity/information content as a constraint in diverse systems. Thus, we wished to examine the generalizability of information as a constraint in systems other than the molecular and genome evolutionary scenarios discussed above. Table 3 shows a wide range of fields of study that utilize information as a parameter, and Table 4 shows some diverse examples where the amount of information and the complexity of a system act as a constraint or cost. Thus, while information has widespread value, it also brings costs, and it is notable that many of the examples listed in Table 4 are related to the increased occurrence of errors and the associated additional resources that are necessary to reduce or avoid these errors. We propose that their consideration might constitute the basis for a generalized theory of errors and their cost in both biological and non-biological systems.

**Table 3:** The importance of information content in diverse systems. The use of information as a parameter in differing fields of study.

Discipline	Parameter
Information theory	Shannon entropy/message length
Signalling games	Complete/incomplete/perfect information
Physics	Physical information
Economics	Information goods
Linguistics	Word/sentence length is related to information content
Ecology	Alpha diversity
Complexity theory	Complexity measures are related to information content
Biology	Genomic information content, organismal complexity

**Table 4:** The importance of information content in diverse systems. Increased information content/complexity may act as a constraint in a variety of different systems, biological and non-biological.

System	Nature of informational/complexity constraint	Consequence
Business	Complexity of business	“Complexity costs“ add financial burden on the business
Healthcare	Complexity of medical treatments	Increased probability of error and consequent detrimental health outcomes [191]
Statistical models	Number of parameters in a model	Greater number of parameters increases the variance of outcome [192]
Messages in communication systems	Message length	Greater message length in communications is costly, leading to the noiseless coding theorum which formalizes message compression [185]
Computer programming	Complexity of code, “feature creep”	Increased production costs
Ecosystem	Biodiversity/number of endemic species	The more biodiverse an ecosystem, the greater the political/economic pressure to preserve it
Biological research	Equation density in a research paper	Reduced citation of paper [193]
Genomics	Quantity and complexity of high throughput data	Analysis costs, *i.e.*, the “bioinformatics bottleneck”
Multicellular animals	Body size	More cells (and so genome copies) proposed to increase cancer risk [194,195,196]
Lateral gene transfer	Complexity of protein complexes	The complexity hypothesis proposes that participation in multi-subunit protein complexes constitutes a barrier to the lateral transfer of informational genes [197]
Organismal evolution	Organismal complexity	Organismal complexity proposed to constrain rate of adaptation [175,198]
Molecular evolution	Genomic information content	Proposed to constrain genetic fidelity [85,86,87,161,164,165] and this work

## 4. Conclusions

This work has examined how the concepts of neutral emergence and information content may explain some key aspects of the genetic code; its robustness to mutational errors and its malleability in some systems, respectively. Empirical and simulation evidence was presented in order to show how these two factors may have influenced genetic code evolution. In addition, the genetic code provides a case study for how these two factors affect evolutionary processes in general. Subsequently, the influence of these factors on other biological traits was explored; many of these are in the process of being explored and elucidated, and it is the purpose of this work to gather together the available evidence from a wide range of biological traits, in order to observe commonalities, using their influence on the genetic code as a starting point and paradigm. Lastly, the role of information content in a variety of non-biological systems was explored, with the finding that information content commonly brings a cost as well as benefit, analogous to its effect in the genome.

## Supplementary Materials

Supplementary materials can be accessed at: http://www.mdpi.com/2075-1729/5/2/1301/s1.
