# Supplementary material for: Genetic Code Evolution Reveals the Neutral Emergence of Mutational Robustness, and Information as an Evolutionary Constraint
Source: Life (Basel). 2015 Apr 24;5(2):1301–32. doi: 10.3390/life5021301 (PMC4500140; doi:10.3390/life5021301)
Supplement: Supplementary file 1 [file life-05-01301-s001.pdf]

Supplementary Materials

Genome sizes were obtained from Genbank and the respective genome sequencing projects. Proteome sizes were determined by counting all codons present in the respectives genomes. Mutation rates were obtained from the references listed. In multicellular eukaryotes the rates are per cell division, the number of germline cell divisions per generation were derived from Drake *et al.* [1], and Hoffman *et al.* [2] Genes Dev 18, 2676 for *A.thaliana*. These numbers are in brackets.

Table S1. Mutation rates of genomes that encode their own replication apparatus.

Genome	Mutation rate per bp per genome replication	Genome size (bp)	Proteome size (codons)	References
Ty1 retrotransposon (RNA) Z48149	0.000058	5925	1595	Drake *et al.* (1998) [3]
Tobacco mosaic virus (RNA) NP597750	0.0000091	6395	2083	Malpica *et al.* (2002) [4]
Rous Sarcoma Virus (RNA) NP056888	0.000046	9392	2129	Drake (1993) [5]
Bovine Leukemia Virus (RNA) AAF97920	0.0000032	8419	2234	Drake *et al.* (1998) [3]
Murine Leukemia Virus (RNA) NC001501	0.000017	8332	2402	Drake (1993) [5]
HIV-1 (RNA) NC001802	0.000029	9181	3069	Drake *et al.* (1998) [3]
Vesicular Stomatitis (RNA) NC001560	0.00031	11161	3536	Drake (1993) [5]
Duck hepadnavirus (RNA) NP039821	0.000027	3027	1143	Pult *et al.* (2001) [6]
Influenza A (RNA) H5N1	0.000074	13410	3557	Drake (1993) [5]
Phage Qbeta (RNA) NC001890	0.0015	4215	1336	Drake (1993) [5]
Phage phi-6 (RNA) NC003714-16	0.0000026	13385	3511	Chao *et al.* (2002) [7]
Poliovirus (RNA) NC002058	0.00011	7440	2209	Drake (1991) [1]
Measles Virus (RNA)	0.000044	15894	5202	Sanjuan *et al.* (2010) [8]
Bacteriophage M13	0.0000072	6407	2099	Drake (1991) [1]
Bacteriophage T4	0.00000024	168903	55846	Drake (1991) [1]
Herpes simplex 1	0.00000018	152260	41810	Drake and Hwang (2005) [9]
Bacteriophage ΦX174	0.000001	5386	2327	Cuevas *et al.* (2009) [10]
Bacteriophage λ	0.000000079	48490	14866	Sanjuan *et al.* (2010) [8]
E.coli	0.0000000054	4639675	1315548	Drake (1991) [1]
Haloferax volcanii	0.0000000011	4010000	1168159	Mackwan *et al.* (2007) [11]
Salmonella enterica	0.0000000034	4755700	1352802	Hudson *et al.* (2003) [12]

Table S1. *Cont.*

Genome	Mutation rate per bp per genome replication	Genome size (bp)	Proteome size (codons)	References
Mycobacterium tuberculosis	0.0000000019	4403840	1352815	Werngren and Hoffner (2003) [13]
Helicobacter pylori	0.0000000066	1667870	496822	Wang *et al.* (2001) [14]
Bacillus anthracis	0.0000000006	5227420	1429238	Vogler *et al.* (2002) [15]
Sulfolobus Sulfolobus acidocaldarius	0.0000000078	2992245	632091	Grogan *et al.* (2001) [16]
Deinococcus radiodurans	0.0000000077	3240000	895466	Kim *et al.* (2004) [17]
Oenococcus oeni	0.0000016	1740517	495710	Marcobal *et al.* (2007) [18]
Leuconostoc mesenteroides	0.00000000218	2075763	616419	Marcobal *et al.* (2007) [18]
Pediococcus pentosaceus	0.00000000263	1832387	543716	Marcobal *et al.* (2007) [18]
Trypanosoma brucei	0.000000001	26000000	7872754	Valdes (1996) [19]
S.cerevisiae	2.2×10^{-10}<br>3.3×10^{-10}<br>5.12×10^{-10}<br>Mean = 3.54×10^{-10}	12070000	2906432	Drake *et al.* (1998) [3]<br>Lynch *et al.* (2008) [20]<br>Lang and Murray (2008) [21]
N.crassa	7.2×10^{-11}	39225835	4784823	Drake *et al.* (1998) [3]
Paramecium tetraurelia	1.94×10^{-11}	72094543	18290912	Sung *et al.* (2012) [22]
Dictyostelium discoideum	2.9×10^{-11}	34204973	7106922	Saxer *et al.* (2012) [23]
Arabidopsis thaliana (35)	2×10^{-10}	93654490	14654490	Ossowski *et al.* (2010) [24]
C.elegans (9)	2.3×10^{-10}<br>2.3×10^{-10}<br>Mean = 2.3×10^{-10}	97000000	10043780	Drake *et al.* (1998) [3]<br>Denver *et al.* (2004) [25]
D.melanogaster (25)	3.4×10^{-10}<br>3.36×10^{-10}<br>Mean = 3.38×10^{-10}	116800000	7103098	Drake *et al.* (1998) [3]<br>Haag-Liautard *et al.* (2007) [12]
Mouse (62)	1.8×10^{-10}	2500000000	15624175	Drake *et al.* (1998) [3]
Homo sapiens (400)	5.0×10^{-11}<br>6.25×10^{-11}<br>4.5×10^{-11}<br>Mean = 5.25×10^{-11}	3200000000	16455036	Drake *et al.* (1998) [3]<br>Nachman and Crowell (2000) [26]<br>Kondrashov (2003) [27]

Reference

1. Drake, J.W. A constant rate of spontaneous mutation in DNA-based microbes. *Proc. Natl. Acad. Sci. USA* **1991**, *88*, 7160–7164.
2. Hoffman, P.D.; Leonard, J.M.; Lindberg, G.E.; Bollmann, S.R.; Hays, J.B. Rapid accumulation of mutations during seed-to-seed propagation of mismatch-repair-defective Arabidopsis. *Genes Dev.* **2004**, *18*, 2676–2685.
3. Drake, J.W.; Charlesworth, B.; Charlesworth, D.; Crow, J.F. Rates of spontaneous mutation. *Genetics* **1998**, *148*, 1667–1686.
4. Malpica, J.M.; Fraile, A.; Moreno, I.; Obies, C.I.; Drake, J.W.; Garcia-Arenal, F. The rate and character of spontaneous mutation in an RNA virus. *Genetics* **2002**, *162*, 1505–1511.
5. Drake, J.W. Rates of spontaneous mutation among RNA viruses. *Proc. Natl. Acad. Sci. USA* **1993**, *90*, 4171–4175.
6. Pult, I.; Abbott, N.; Zhang, Y.-Y.; Summers, J. Frequency of spontaneous mutations in an avian hepadnavirus infection. *J. Virol.* **2001**, *75*, 9623–9632.
7. Chao, L.; Rang, C.U.; Wong, L.E. Distribution of spontaneous mutants and inferences about the replication mode of the RNA bacteriophage phi6. *J. Virol.* **2002**, *76*, 3276–3281.
8. Sanjuan, R.; Nebot, M.R.; Chirico, N.; Mansky, L.M.; Belshaw, R. Viral mutation rates. *J. Virol.* **2010**, *84*, 9733–9748.
9. Drake, J.W.; Hwang, C.B.C. On the mutation rate of herpes simplex virus type 1. *Genetics* **2005**, *170*, 969–970.
10. Cuevas, J.M.; Duffy, S.; Sanjuan, R. Point mutation rate of Φ174. *Genetics* **2009**, *183*, 747–749.
11. Mackwan, R.R.; Carver, G.T.; Drake, J.W.; Grogan, D.W. An unusual pattern of spontaneous mutations recovered in the halophilic archaeon *Haloferax volcanii*. *Genetics* **2007**, *176*, 697–702.
12. Haag-Liautard, C.; Dorris, M.; Maside, X.; Macaskill, S.; Halligan, D.L.; Charlesworth, B.; Hudson, R.E.; Bergthorsson, U.; Ochman, H. Transcription increases multiple spontaneous point mutations in *Salmonella enterica*. *Nucleic Acids Res.* **2003**, *31*, 4517–4522.
13. Werngren, J.; Hoffner, S.E. Drug-susceptible *Mycobacterium tuberculosis* Beijing genotype does not develop mutation-conferred resistance to rifampin at an elevated rate. *J. Clin. Microbiol.* **2003**, *41*, 1520–1524.
14. Wang, G.E.; Wilson, T.J.M.; Jiang, Q.; Taylor, D.E. Spontaneous mutations that confer antibiotic resistance in *Helicobacter pylori*. *Antimicrob. Agents Chemother.* **2001**, *45*, 727–733.
15. Vogler, A.J.; Busch, J.D.; Percy-Fine, S.; Tipton-Hunton, C.; Smith, K.L.; Keim, P. Molecular analysis of rifampin resistance in *Bacillus anthracis* and *Bacillus cereus*. *Antimicrob. Agents Chemother.* **2002**, *46*, 511–513.
16. Grogan, D.W.; Carver, G.T.; Drake, J.W. Genetic fidelity under harsh conditions: analysis of spontaneous mutation in the thermoacidophilic archaeon *Sulfolobus acidocaldarius*. *Proc. Natl. Acad. Sci. USA* **2001**, *98*, 7928–7933.
17. Kim, M.; Wolff, E.; Huang, T.; Garibyan, L.; Earl, A.M.; Battista, J.R.; Miller, J.H. Developing a genetic system in *Deinococcus radiodurans* for analyzing mutations. *Genetics* **2004**, *166*, 661–668.
18. Marcobal, A.M.; Sela, D.A.; Wolf, Y.I.; Makarova, K.S.; Mills, D.A. Role of hypermutability in the evolution of the genus Oenococcus. *J. Bacteriol.* **2008**, *190*, 564–570.

19. Valdes, J.; Taylor, M.C.; Cross, M.A.; Ligtenberg, M.J.L.; Rudenko, G.; Borst, P. The viral thymidine kinase gene as a tool for the study of mutagenesis in *Trypanosoma brucei*. *Nucleic Acids Res.* **1996**, *24*, 1809–1815.
20. Lynch, M.; Sung, W.; Morris, K.; Coffey, N.; Landry, C.R.; Dopman, E.B.; Dickinson, W.J.; Okamoto, K.; Kulkarni, S.; Hartl, D.L. A genome-wide view of the spectrum of spontaneous mutations in yeast. *Proc. Natl. Acad. Sci. USA* **2008**, *105*, 9272–9277.
21. Lang, G.I.; Murray, A.W. Estimating the per-base-pair mutation rate in the yeast *Saccharomyces cerevisiae*. *Genetics* **2008**, *178*, 67–82.
22. Sung, W.; Tucker, A.E.; Doak, T.G.; Choi, E.; Thomas, W.K.; Lynch, M. Extraordinary genome stability in the ciliate *Paramecium tetraurelia*. *Proc. Natl. Acad. Sci. USA* **2012**, *109*, 19339–19344.
23. Saxer, G.; Havlak, P.; Fox, S.A.; Quance, M.A.; Gupta, S.; Fofanov, Y.; Strassman, J.E.; Queller, D.C. Whole genome sequencing of mutation accumulation lines reveals a low mutation rate in the social amoeba *Dictyostelium discoideum*. *PLoS ONE* **2012**, *7*, e46759.
24. Ossowski, S.; Schneeberger, K.; Lucas-Lledo, J.I.; Warthmann, N.; Clark, R.M.; Shaw, R.G.; Weigel, D.; Lynch, M. The rate and spontaneous molecular spectrum of spontaneous mutations in *Arabidopsis thaliana*. *Science* **2010**, *327*, 92–94.
25. Denver, D.R.; Morris, K.; Lynch, M.; Thomas, W.K. High mutation rate and predominance of insertions in the *Caenorhabditis elegans* nuclear genome. *Nature* **2004**, *430*, 679–682.
26. Nachman, M.W.; Crowell, S.L. Estimate of the mutation rate per nucleotide in humans. *Genetics* **2000**, *156*, 297–304.
27. Kondrashov, A.S. Direct estimates of human per nucleotide mutation rates at 20 loci causing Mendelian diseases. *Hum. Mutat.* **2003**, *21*, 12–27.
